# Does appreciative inquiry decrease false positive diagnosis during leprosy case detection campaigns in Bihar, India? An operational research study

**DOI:** 10.1371/journal.pntd.0007004

**Published:** 2018-12-21

**Authors:** Ashish Nareshkumar Wagh, Shivakumar Mugudalabetta, Nimer Ortuno Gutierrez, Krishnamurthy Padebettu, Ajay Kumar Pandey, Bijoy Kumar Pandey, Mahalakshmy Thulasingam, Srinath Satyanarayana, Amol Dongre

**Affiliations:** 1 Damien Foundation India Trust, Bihar, India; 2 VDamien Foundation, Bruxelles, Belgium; 3 State Leprosy Officer, Bihar, India; 4 Jawaharlal Institute of Postgraduate Medical Education & Research (JIPMER), Puducherry, India; 5 Center for Operational Research, International Union against Tuberculosis and Lung Disease, Paris, France; 6 Sri Manakula Vinayagar Medical College and Hospital, Puducherry, India; Hospital Infantil de Mexico Federico Gomez, UNITED STATES

## Abstract

**Background:**

India contributes ~60% to the global leprosy burden. The country implements 14-day community-based leprosy case detection campaigns (LCDC) periodically in all high endemic states. Paramedical staff screen the population and medical officers of primary health centres (PHCs) diagnose and treat leprosy cases. Several new cases were detected during the two LCDCs held in September-2016 and February-2018. Following these LCDCs, a validation exercise was conducted in 8 Primary health centres (PHCs) of 4 districts in Bihar State by an independent expert group, to assess the correctness of case diagnosis. Just before the February 2018 LCDC campaign, we conducted an “appreciative inquiry” (AI) involving the health care staff of these 8 PHCs using the 4-D framework (Discovery-Dream-Design-Destiny).

**Objectives:**

To assess whether the incorrect case diagnosis (false positive diagnosis) reduced as a result of AI in the 8 PHCs between the two LCDC conducted in September-2016 and February-2018.

**Methodology/principal findings:**

A three-phase quantitative-qualitative-quantitative mixed methods research (embedded design) with the two validation exercises conducted following September-2016 and February-2018 LCDCs as quantitative phases and AI as qualitative phase. In September-2016 LCDC, 303 new leprosy cases were detected, of which 196 cases were validated and 58 (29.6%) were false positive diagnosis. In February-2018 LCDC, 118 new leprosy cases were detected of which 96 cases were validated and 22 cases (23.4%) were false positive diagnosis. After adjusting for the age, gender, type of cases and individual PHCs fixed effects, the proportion of false positive diagnosis reduced by -9% [95% confidence intervals (95%CI): -20.2% to 1.7%, p = 0.068]

**Conclusion:**

False positive diagnosis is a major issue during LCDCs. Though the decline in false positive diagnosis is not statistically significant, the findings are encouraging and indicates that appreciative inquiry can be used to address this deficiency in programme implementation.

## Author summary

India is the highest leprosy burden country in the world. Government of India’s National Leprosy Eradication Programme (NLEP) launched Leprosy Case Detection Campaign (LCDC)—an active community-based case detection campaign—in 2016 in all high burden areas to detect undiagnosed cases. Following these LCDC, a small validation exercise was conducted in 8 Primary health centres (PHCs) in Bihar State by an independent expert group, to assess the correctness of case diagnosis. It found that ~30% of the cases detected were not true cases, but false positive diagnosis. To reduce false positive diagnosis in the subsequent round of LCDC in 2018, an “appreciative inquiry” involving the health care staff of these 8 PHCs using the 4-D framework (Discovery-Dream-Design-Destiny) was done. In 2018 LCDC, the false positive diagnosis decreased to ~23%. After adjusting for the differences in the patient and health facility characteristics, the decline in false positive diagnosis was estimated to be about 9%. This study shows that false positive diagnosis was a major issue during LCDCs and that appreciative inquiry can be used to address this deficiency in programme implementation.

## Introduction

Leprosy is a chronic infectious disease caused by the bacteria—*Mycobacterium Leprae*. It usually affects the peripheral sensory nerves and has a wide range of clinical manifestations. The disease is characterized by long incubation period generally 5–7 years. Leprosy is completely curable with 6–12 months of multidrug therapy. Early diagnosis and treatment of cases is the most effective way of halting transmission and eliminating leprosy from the community [1].

India is the highest leprosy burden country in the world. In 2016, ~135,000 new cases of leprosy were detected by the Government of India’s National Leprosy Eradication Programme (NLEP). This constituted about 66% of total leprosy cases detected in the world in that year [2,3]. In India, in 2016, the Annual New Case Detection Rate (ANCDR) was 9.71 cases per 100,000 population and Prevalence Rate (PR) was 0.66 per 10,000 population. ANCDR and PR have been showing stable trends since 2006. The other leprosy indicator related to the child cases (number and proportion of new cases aged <15 years) is also relatively high indicating on-going transmission in the community [4,5]. The major source of transmission of infection in the community are the hidden undiagnosed and untreated cases. Hence, in order to detect these cases, NLEP introduced yearly Leprosy Case Detection Campaign (LCDC)—community based active case finding campaign—in 2016 in high endemic states [6].

Bihar, a state in the eastern part of India (population of 113 million), is one of the highest leprosy burden states in the country. It is reporting 16,000 to 20,000 new cases of leprosy every year since 2005 (15–20% of the cases in the country). In 2016, Bihar reported 16,185 new cases of leprosy. LCDC was carried out in Bihar in 2016 in 20 out of 38 districts and this yielded 4517 new cases of leprosy [2]. The campaign was organised for 14 days from 5–18 September 2016.

Damien Foundation India Trust (DFIT), is a charitable Non-Governmental Organization working for leprosy and tuberculosis control in Bihar. DFIT provides technical support to NLEP in planning, implementing, monitoring, and evaluation. DFIT organised a validation exercise in collaboration with the State Leprosy Programme Officer, Bihar. The validation exercise was carried out by an independent expert group to assess the quality of diagnosis among the cases detected during the campaign. Two blocks in each of the four districts- Nalanda, Sitamarhi, Gopalganj and Araria (which reported highest number of cases during LCDC) were selected for validation. It was found that about 30% of cases detected during LCDC were wrongly diagnosed as leprosy cases (false positive cases). False positive diagnosis leads to unnecessary medication, causes stigma, isolation, loss of employment and discrimination that can lead to considerable mental trauma and agony in the patients and their families [7,8]. In addition, it also discredits the LCDC campaign. Thus, there was an urgent need to understand the reasons for false positive diagnosis and undertake suitable corrective measures to address this issue.

Diagnosis of leprosy requires specific clinical expertise. Anecdotal discussions with the programme staff indicated that with a general decline in leprosy cases over the last few decades, there has been a decline in the clinical expertise within the public health system to diagnose leprosy due to retirement of trained leprosy personnel without new recruitments, inadequate trainings, transfer of existing leprosy trained workforce to other public health programmes etc. In Indian public health programme settings, the traditional approach for problem-solving is generally characterised by fault finding and penalization. In contrast, we wanted to test a flexible and friendly approach for reducing false positive diagnosis.

Appreciative Inquiry (AI)—is a philosophical approach to organizational learning, change management and research. It is a process which shifts the focus of programme or organization from problem identification, defensiveness and denial of facts towards discovery of programme strengths and building on what works well in the given setting and context [9]. This approach has been found effective in improving obstetric referral system in Cambodia [10], improvement of community-based mental health services [11], improvement in nursing care in hospital setting in the United Kingdom [12], and development of better health care work environment in NHS [13]. AI offers a framework which positively influences organizational growth by generating common goals and actions to be achieved by the programme staff [11]. It is emerging as a promising approach for staff motivation and programme sustainability in public health programmes in low and middle-income countries.

Therefore, in 2017–2018, we conducted an operational research study to assess whether AI with health staff reduces the number (and proportion) of false positive diagnosis of leprosy cases during the LCDC in February 2018 when compared to LCDC in September 2016.

## Methods

### Study design

**Fig 1:**
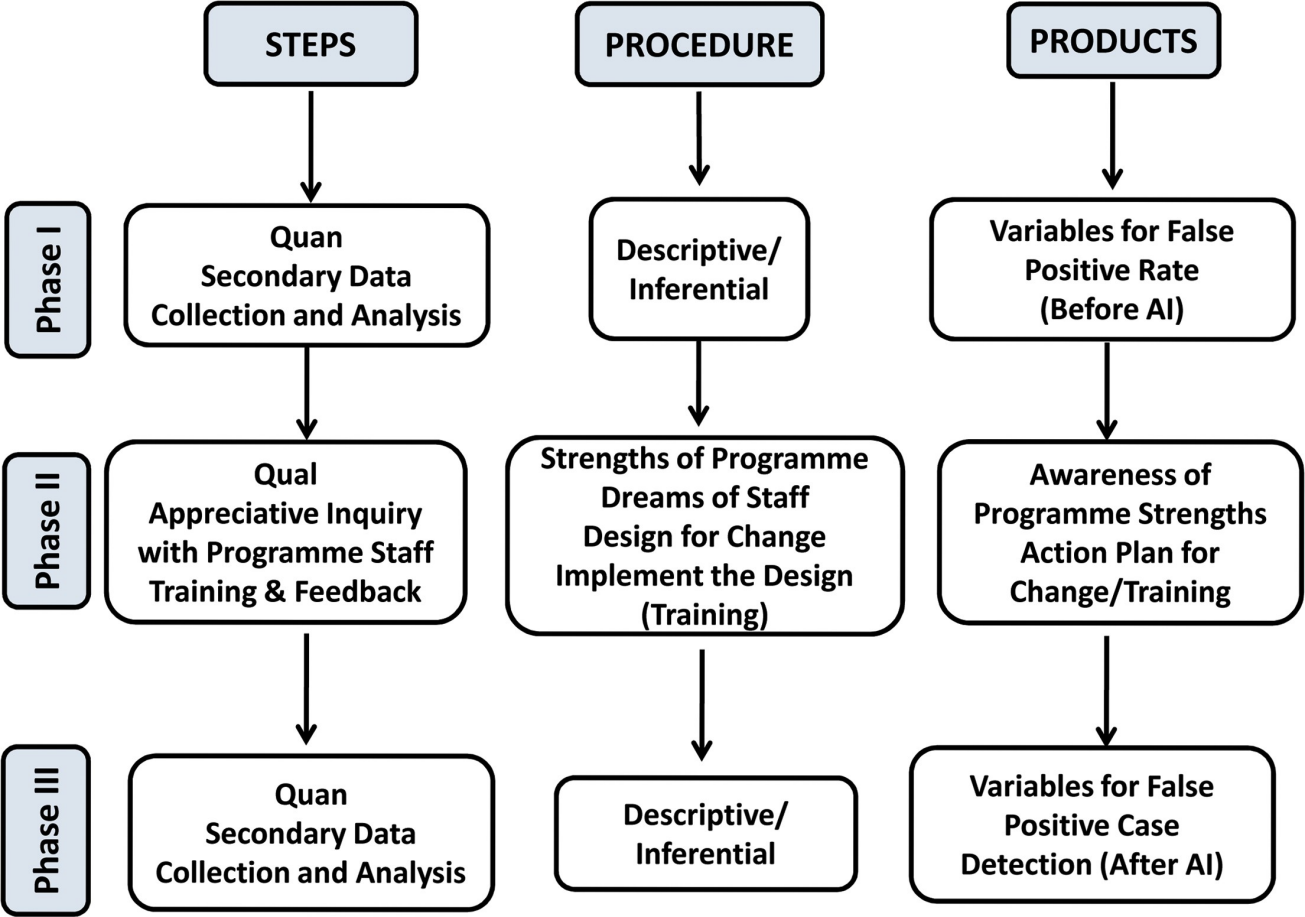
Visual Diagram of study design, data collection and products for assessing whether appreciative inquiry will reduce false positive leprosy diagnosis in Bihar, India (2016–2018).

This is a three-phase mixed methods study (embedded design). The quantitative part contained a before-after study design and the qualitative intervention comprised of appreciative inquiry (**Fig 1**).

### Setting

#### General

Leprosy is one of the major health problems in Bihar, with the State contributing 15% to 20% of the total new cases in India. While India declared elimination of leprosy at country level (prevalence <1 per 10000 population) in 2000, Bihar declared elimination only in 2013. Leprosy programme was integrated into general health services in 2005 irrespective of elimination status at the State level. It was observed that dedicated NLEP staff were mainly involved in management of leprosy cases even after this integration. Majority of vertical NLEP staff have been retiring from service with no new recruitments. Therefore, the management of leprosy and its complications is fast becoming a challenge with staff of the General Health System not adequately trained in diagnosis and management of leprosy cases.

#### Study site

**Fig 2:**
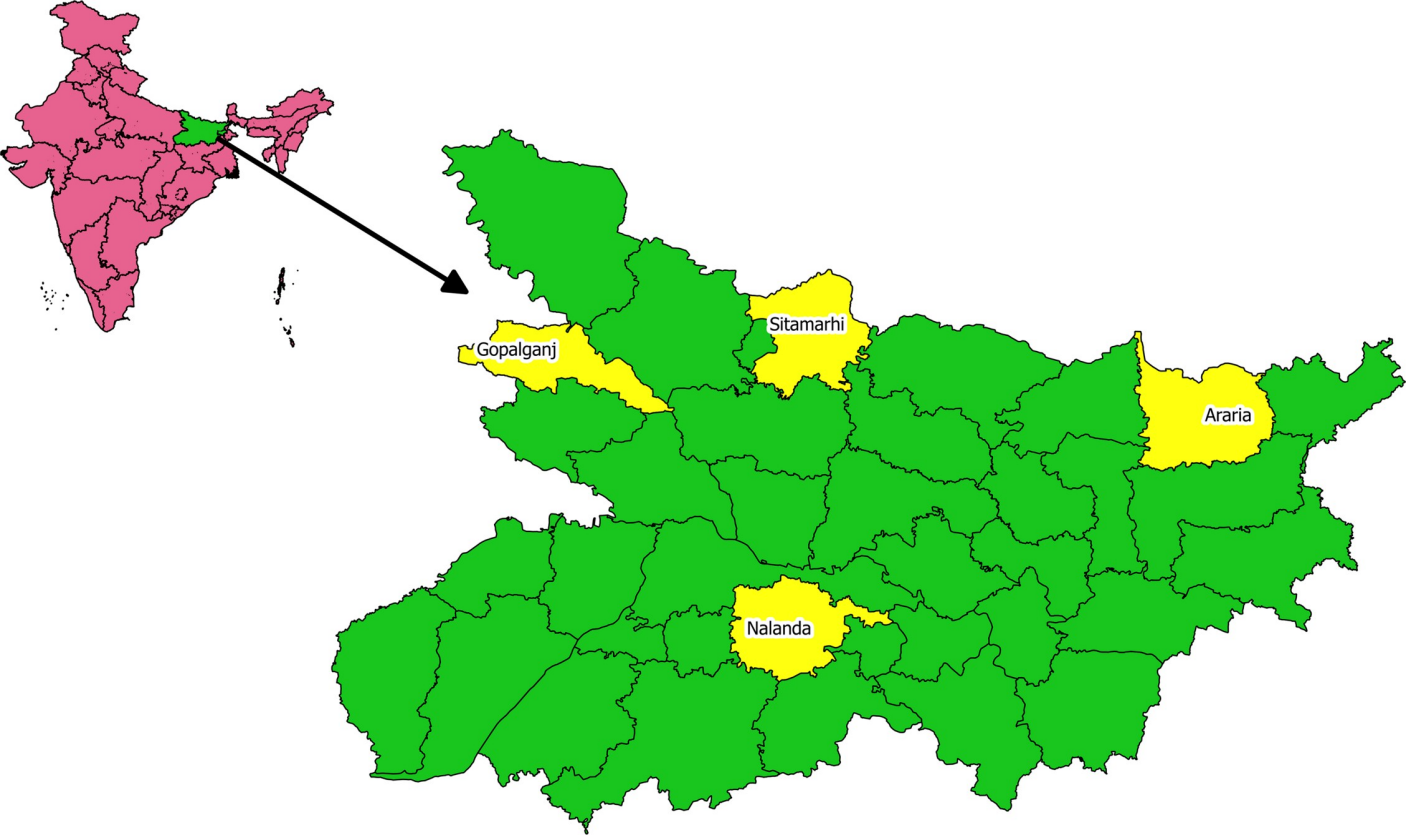
Map of Bihar State showing 4 districts involved in the study to assess whether appreciative inquiry will reduce false positive leprosy diagnosis in Bihar, India (2017–2018). (Source for India admin shape files– http://diva-gis.org/download) free and open source. Software used to create Fig 2 –QGIS (previously known as Quantum GIS) is a free and open source cross platform desktop geographic information system (GIS) application that supports viewing, editing and analysis of geospatial data).

The study was conducted in eight blocks (Nagarnausa, Giriyak, Batnaha, Dumra, Forbesganj, Sikty, Thawe and Hatuwa) of four districts (Nalanda, Sitamarhi, Araria and Gopalganj) located in different parts of Bihar (**Fig 2**). These blocks were selected for the validation study in 2016 as they had the largest number of cases detected during the LCDC in that year. Total population in the 8 blocks in the 4 districts covered through validation was ~ 2 million.

#### Diagnosis and treatment of leprosy during LCDC

There are 534 PHCs in 38 districts of Bihar. Re-orientation training for diagnosis and treatment of leprosy for the medical officers was done at district level and for Accredited Social Health Activists (also called ASHAs—female health volunteers from the community) it was held at PHC level once every year. An ASHA and one male volunteer from the community were identified for every 1000 population and trained in examination of whole body and identifying skin lesions indicative of leprosy. They are responsible to cover 1000 population in 14 days and report all the persons with suspected lesions to the primary health centre for diagnosis of leprosy and further management. At every household, they inform the household members about the purpose of their visit and request the household members to undergo a general physical examination to identify any unusual hypo pigmented skin patches on their body. Male volunteers examine the male members of the family and ASHAs examine the female members of the family. If any skin lesions are observed, then such persons are referred to the concerned primary health centers for further evaluation. At primary health centre, Medical Officers are responsible for leprosy diagnosis. The Medical Officers diagnose, classify and initiate treatment for leprosy based on the diagnostic and treatment criteria given in **Table 1**.

**Table 1:** Classification, treatment regimen & treatment outcomes for leprosy as per national leprosy eradication programme in India.

Characteristic	PB (Pauci Bacillary)	MB (Multi Bacillary)
**For diagnosis**		
Skin lesions	1–5 lesions with definite loss of sensation	6 and above with definite loss of sensation
Peripheral nerve involvement	No nerve/ only one nerve	More than one nerve
Skin smear	Negative at all sites	Positive at any site
**For treatment**		
Drugs and regimen	Rifampicin (once monthly)	Rifampicin (once monthly) Clofazimine (both once monthly and daily) Dapsone (daily)
	Dapsone (daily)	
	
Criteria for release from treatment (RFT)	Completion of 6 monthly pulses in 9 consecutive months	Completion of 12 monthly pulses in 18 consecutive months

### Study population

The study population included all leprosy cases detected during LCDCs in September 2016 and in February 2018 in 8 blocks of 4 districts and validated by the DFIT team. For the qualitative part, the following staff were invited to the AI meeting: at least one Medical Officer from each PHC, Block Community Mobiliser, Block Health Manager, District Nucleus Team, Communicable Disease Officer.

### Method used by DFIT for validating cases detected during LCDC

The validation was undertaken within four weeks of LCDC. In 2016, four teams were formed for the exercise, each consisting of a Medical Officer, a supervisor with more than 10 years of experience in leprosy diagnosis from the State level and another supervisor from the district nucleus team. This team attempted to validate all the new leprosy patients diagnosed during LCDC and assessed whether these cases were true positive cases or false positive cases using the same clinical diagnostic criteria given in Table 1. In this process, they also collected socio-demographic and clinical data from the patients and noted their findings using a structured data collection case sheet. Cases were examined either at the primary health centres or at the patients’ residences.

### Data variables, sources of data and data collection

For the quantitative part, the individual patient wise data of all cases diagnosed as leprosy during LCDC conducted in 2016 & 2018 in these 8 blocks were available with the State NLEP Office in Patna. The patient wise data collected during validation exercise in 2016 & 2018 was available at the DFIT office in Patna. The principal investigator (ANW) obtained these data for its usage in this study. The patient wise data contained information on the name of the patient, age, sex, type of case (PB or MB), PHC, district, block and disability grading in accordance with the NLEP guidelines.

We followed the Appreciative Inquiry framework to plan the intervention One appreciative inquiry meeting was held in each of the four districts in the month of November-December 2017. Formal permissions from the district health authorities were obtained for this meeting. It was facilitated as a group activity. A total of 43 personnel belonging to to various health cadre as mentioned above participated in these meetings (>90% participation). The participants were informed about the purpose of the meeting and were oriented to the philosophy of AI at the time of the meeting. Each meeting had four sequential phases—Discovery, Dream, Design and Destiny (4D)—as per the AI framework (**Box 1**)**. Discovery:** After creating a climate of open exchange, this step was implemented to explore the strengths and positive experiences on what is working well in the programme from each of the participant. **Dream:** This phase of the meeting was facilitated on the broad themes emerging in the ‘discovery’ phase to challenge the status-quo and dream for the better programme achievements. The participants were asked to share their suggestions to improve the programme activities further. **Design**: In this phase, participants were asked to design the action plan for improvement or change in the desired direction based on the collective dream. **Destiny**: In this phase pre-conditions crucial for change or improvement to happen were discussed.

**Box 1: Appreciative Inquiry (AI) procedure followed in the study to assess whether AI reduces false positive leprosy diagnosis in Bihar, India (2017–2018)**

- The concept and methodology of Appreciate Inquiry was explained to the State Programme Officer (SPO) for which approval was obtained.
- Official letters were sent from SPO to Civil Surgeons (Head of district) and Communicable Disease Officers (CDO) responsible for NLEP in the district, Medical Officers and key NLEP staff of 04 districts for their information and cooperation.
- AI meeting was arranged at district Head Quarters. Meeting was organized in big hall with chairs arranged in circular manner so that all the participants were facing each other.
- Principal Investigator briefed about the concept and methodology of AI and its 4D framework to all participants.
- For the first component of AI–Discovery, all the participants were given colour coded cards and was asked to pen down their thoughts about their strengths and positive experiences on what is working well in the programme among their own capacity of work area. Everyone was asked to share their thoughts about their strengths.
- Based on Discovery ANW briefed about the second component of AI- Dream and asked all the participants to imagine, what is their dream to achieve the desired outcomes within the programme in the present situation. Everyone was asked to share their dreams.
- Again, the ANW briefed about the third component of AI–Design and asked the participants. Based on their dreams everyone was asked to design an action plan for the improvement or to bring change in their desired area of work according to their individual capacity of work which can improve the results of programme. Everyone was asked to share their plans of action to improve the programme outcomes.
- After that ANW briefed about the Destiny component of AI with the District programme officer i.e. CDO and discussed with him the recommendations of the participants to be implemented into the programme to achieve better results during the next active search case detection campaign.

The district leprosy officer (Communicable Disease Office) was involved and briefed about the AI approach and its philosophy to seek his full co-operation in the improvement process.

In ‘Appreciative Inquiry’ approach (AI), the questions pertained to the following: Experience—based on your experience, what is the current status of the leprosy programme?; Opinion—What is your opinion on the current status of the leprosy programme?; Suggestions—What could be the ways to improve the current status of the programme?; Discover—Tell me that high point in the leprosy programme which makes you feel high; Dream—What do you wish to improve in leprosy programme in the future?[14]

### Analysis and statistics

Quantitative: All quantitative data analysis was done using EpiData [version 2.2.2.183, EpiData Association, Odense, Denmark] and Stata [Version 15, StataCorp, College Station, Texas, United States]. The demographic and clinical characteristics has been summarized using frequencies and percentage. We compared the demographic and clinical characteristics of patients detected during LCDC and patients reached during validation in 2016 and 2018 using Chi-square test. We used log binomial models with robust standard error estimates to obtain the adjusted differences in the proportion of false positive cases (in those validated) between 2016 and 2018 after adjusting for the differences in age, sex, type of case and the PHCs from which these cases were detected. A P-value < 0.05 was considered for statistical significance. Qualitative: For the analysis of qualitative interview we used the AI framework. The issues that emerged from the four meetings were grouped into three broad themes. The themes were similar to the Discovery, Dream, Design concept of the AI framework. The themes were ‘strengths of the program’, ‘imagined future outcome of the program’, ‘suggestions to improve the program in future’[14]. The similar issues within a theme was grouped into categories. Two investigators did the analysis independently. grouped the issues into these themes. Any discrepancies were sorted out by discussion. The final analysis was finally reviewed by another investigator.

### Ethics approval

We obtained ethics approval for this study from the Ethics Advisory Group of the International Union Against Tuberculosis and Lung Disease, Paris, France and from the ethics review board of the Sri Manakula Vinayagar Medical College and Hospital Pondicherry, India. We obtained administrative approvals for conducting this study from the State and the four District Leprosy Officers. For the quantitative component of the study, which involved the retrospective review of patient records, we got a waiver from obtaining informed consent from patients. However, we obtained written informed consent from all the participants who were part of the Appreciative inquiry meetings.

## Results

### Quantitative (phase 1)

**Fig 3:**
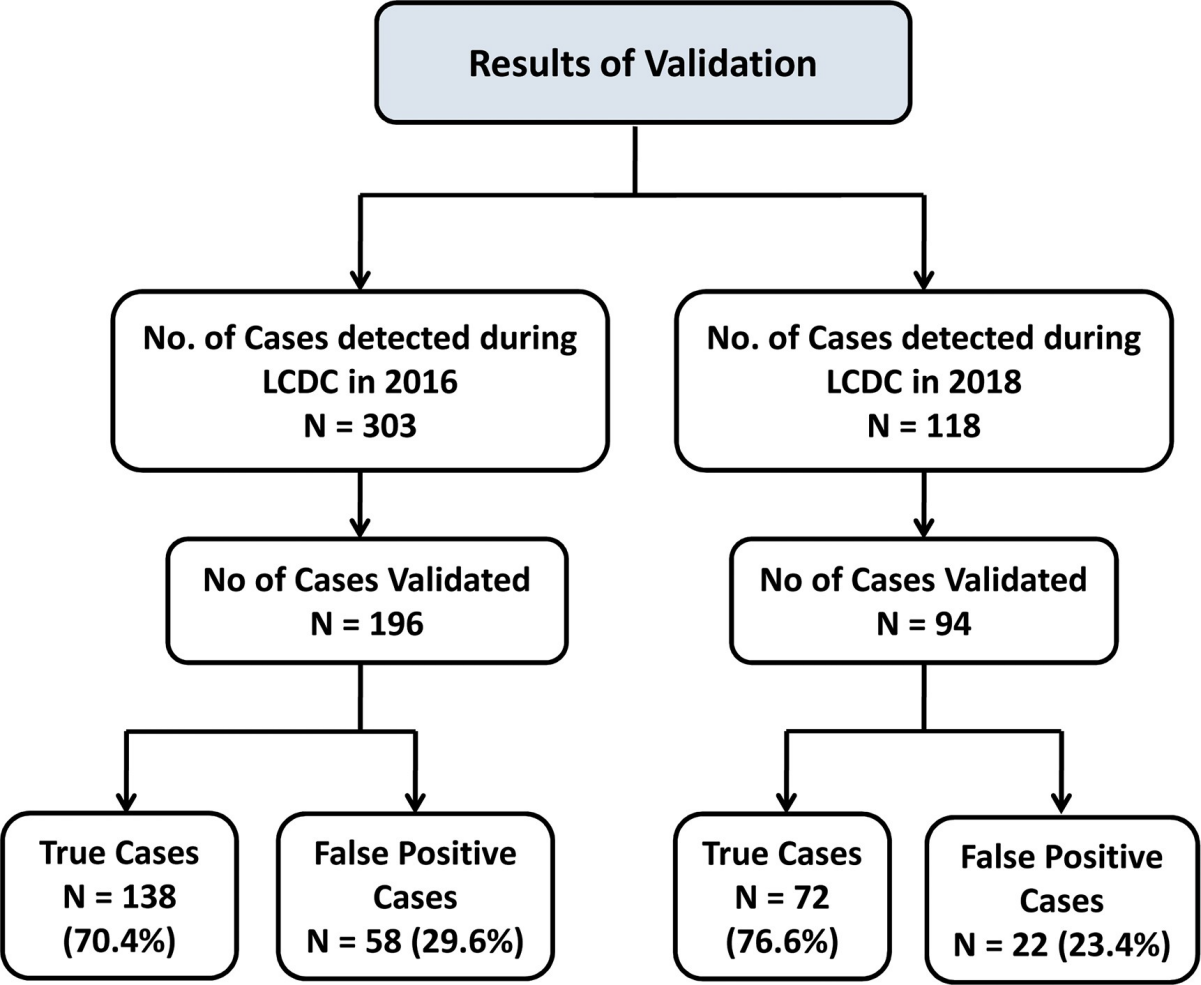
Validation of leprosy cases detected during 2016 and 2018 by DFIT in 8 blocks of 4 districts of Bihar, India 2016–2018.

In 2016, 303 leprosy cases were detected during LCDC in the 8 PHCs of which 196 cases could be validated. Of those validated, 58 (29.6%) were false positive cases **(Fig 3)**. The proportion of cases validated when compared to detected cases did not differ by age, gender and type of leprosy cases. However, proportion validated differed across the 8 PHCs (**Table 2**).

**Table 2:** Characteristics of the patients diagnosed during Leprosy case detection campaign (LCDC) and cases validated by DFIT in 8 blocks of 4 districts in Bihar, India (2016–2018).

Characteristics	2016			2018		
	LCDC cases	Validated cases	Chi-square test P-value	LCDC cases	Validated cases	Chi-square test P-value
**Total No. of Cases**	303	196		118	94	
	N (%)	N (%)		N (%)	N (%)	
**Age (in years)**						
<6	3 (1)	2 (1)	0.199	2 (2)	2 (2)	0.097
6–14	48 (16)	37 (19)		13 (11)	12 (13)	
15–30	115 (38)	75 (38)		44 (37)	30 (32)	
>30	137 (45)	82 (42)		59 (50)	50 (53)	
**Gender**						
Male	142 (47)	86 (44)	0.158	64 (54)	46 (49)	0.022
Female	161 (53)	110 (56)		54 (46)	48 (51)	
**Type of Cases**						
Pauci bacillary	232 (77)	153 (78)	0.406	76 (64)	65 (69)	0.033
Multi bacillary	71 (23)	43 (22)		42 (36)	29 (31)	
**Districts**						
Nalanda	50 (17)	38 (20)	<0.001	16 (14)	16 (17)	0.015
Sitamarhi	73 (24)	47 (24)		41 (35)	33 (35)	
Araria	139 (46)	75 (38)		51 (43)	35 (37)	
Gopalganj	41 (13)	36 (18)		10 (7)	10 (11)	
**Primary Health Centres**						
1	26 (9)	17 (9)	0.001	6 (5)	4 (4)	0.009
2	24 (8)	21 (11)		35 (30)	29 (31)	
3	39 (13)	26 (13)		28 (24)	18 (19)	
4	34 (11)	21 (11)		7 (6)	7 (7)	
5	91 (30)	45 (23)		1 (1)	1 (1)	
6	48 (16)	30 (15)		9 (8)	9 (10)	
7	19 (6)	17 (8)		23 (19)	17 (18)	
8	22 (7)	19 (9)		9 (8)	9 (10)	

### Qualitative (phase 2)

As planned, four AI meetings were held, one in each district. The themes that emerged during these meetings pertaining to discovery, dream, design is summarised in **(Table 3)**. The major strengths of the programme were availability of manpower and infrastructure, availability of commodities for management of leprosy, administrative support from government and other external sources. The imagined future outcome of the program was leprosy free society without stigma, discrimination and a well-informed society. The proposed action plan to achieve the future outcomes included reorientation training of all the programme staff, financial and administrative support, improved intersectoral co-ordination, better referral system, strengthening supervision and monitoring, health education of the community and implementation of the social welfare schemes.

**Table 3:** Results and themes derived from appreciative inquiry prior to leprosy case detection campaign in Bihar, India (November 2017).

**Theme 1: Strengths of the program** **Availability of manpower and infrastructure** • Presence of ASHA workers in the program. • Availability of Leprosy Tertiary Centers—TLMI Muzaffarpur and MLCU Rudrapura. **Availability of commodities for Leprosy management** • Availability of Multi Drug Therapy at the districts/PHCs. • Availability of T. Prednisolone for treatment of reactions-which causes disability in leprosy. • Availability of Micro Cellular Rubber footwear and Self-Care kits. **Administrative support** • Support of NLEP Consultant for the programme. • Support of State Officials (State Leprosy Officer). • Support of local NGO (DFIT) for monitoring and supervision activities. **Existence of constructive schemes** • Disability Pensions for persons affected by leprosy. • Incentives for ASHAs for case detection and treatment completion.
**Theme 2: Imagined future outcome of the program** **Leprosy free India** • No person should suffer from leprosy in India. • No child should develop disability due to Leprosy. • The Lepra bacilli transmission in India should end. **Society without stigma against leprosy** • Every citizen in India takes the social responsibility of eliminating leprosy • Better opportunities for persons affected by leprosy. • No person should suffer stigma due to leprosy. **Well informed society** • People are aware of symptoms of leprosy and where to seek care.
**Theme 3: Action to achieve the aspiration (Suggestions to better the program in future)** **Re-orientation training** • Re-orientation training of staff is required before every campaign. • Training of support staff also is necessary. • Training on maintenance of records and reports of programme. • ASHAs should be trained and motivated by highlighting their role. **Financial and administrative support** • Timely incentives to ASHAs. • Enough fund allocation for the programme especially for supervision and monitoring. **Improve Inter-sectoral co-ordination** • Actively involve other departments such as social welfare, education, agriculture, etc. • Involve Anganwadi Workers especially in places where ASHAs are not available. • Involvement of local and national NGOs in the programme. **Better referral system Leprosy** • Primary, Secondary and Tertiary level referral system should be interlinked with regular feedback. **Strengthen supervision and monitoring** • Supervision and monitoring to done at all levels. • Vehicle support should be provided to districts for supervision and monitoring. • Program activities and results should be regularly reviewed at all levels. • Regular review and updating the records and reports. • One nodal person should be responsible at all levels for the programme. **Health education** • Increase the information, education & communication activities in villages. • Advertisements in mass media to generate interest within the people. • Design innovative and culturally sensitive awareness campaigns. • Use of technology and social media (WhatsApp and Facebook) to spread awareness. • Leprosy should be included in school textbooks. • Political leaders should speak about leprosy in different forums. **Implementation of welfare schemes** • Schemes should be launched by government for upliftment of persons affected. • Allocation of government and private jobs/incentives to the persons affected.

### Quantitative (phase 3)

In 2018, 118 leprosy cases were detected during LCDC in the same 8 PHCs—62% decline in the number of new cases diagnosed when compared to LCDC conducted in 2016. Of the 118 cases detected, 94 cases were validated. Of those validated, 22 cases (23.4%) were false positive cases (**Fig 3**). The proportion of cases validated differed from the cases detected in LCDC by gender, type of cases, across districts and PHCs (**Table 2**).

### Change in false positive cases among validated cases between 2016 and 2018

After adjusting for the age, gender, type of cases and individual PHCs fixed effects, the prevalence ratio of false positive cases between 2016 and 2018 was 0.67 (95% CI: 0.44–1.03, p = 0.068) indicating a 33% decline in the relative prevalence of false positive cases in 2018 across 8 PHCs when compared to 2016 (**Table 4**). From the coefficients of the model used to derive the adjusted prevalence ratios, the adjusted estimated decline in the proportion of false positive cases between 2016 and 2018 was -9% (95% CI: -20.2% to 1.7%). The proportion of false positive cases across PHCs varied widely and it ranged from 3.6% to 46% with the false positive cases in some PHCs were almost 3–4 times higher than the others.

**Table 4:** Association of appreciative inquiry and patient characteristics with false positive diagnosis of leprosy during Leprosy case detection campaign in Bihar, India (2016–2018).

Characteristics of Diagnosis	Total number of Patients validated (N = 290)	False Positive diagnosis		Unadjusted		Adjusted		
	N	N	(%)	PR*	(95%CI)	PR*	(95%CI)	P-value
**Appreciative Inquiry**								
Before (2016)	196	58	(29.6)	Ref	-	Ref	-	
After (2018)	94	22	(23.4)	0.89	(0.72–1.10)	0.67	(0.44–1.03)	0.068
**Age (yrs)**								
<6	4	2	(50.0)	2.28	(0.80–6.49)	1.54	(0.69–3.44)	0.291
6–14	49	8	(16.3)	0.74	(0.35–1.54)	0.69	(0.34–1.39)	0.306
15–30	105	23	(21.9)	Ref		Ref		
>30	132	47	(35.6)	**1.16**	**(1.05–2.49)**	**1.50**	(0.99–2.26)	0.054
**Gender**								
Male	132	38	(28.8)	Ref		Ref		
Female	158	42	(26.6)	0.92	(0.63–1.34)	1.00	(0.70–1.45)	0.960
**Type of Cases**								
Pauci Bacillary	218	61	(28.0)	Ref		Ref		
Multi Bacillary	72	19	(26.4)	0.94	(0.60–1.46)	0.83	(0.54–1.26)	0.395
**PHC**								
1	30	3	(10.0)	Ref		Ref		
2	50	17	(34.0)	**3.40**	**(1.08–10.6)**	3.85	(1.22–12.10)	**0.021**
3	63	29	(46.0)	**4.60**	**(1.51–13.9)**	4.94	(1.65–14.75)	**0.004**
4	28	5	(17.9)	1.78	(0.46–6.80)	1.96	(0.52–7.36)	0.314
5	18	3	(16.7)	1.66	(0.37–7.41)	2.27	(0.52–9.89)	0.272
6	26	11	(42.3)	**4.23**	**(1.31–13.57)**	5.38	(1.69–17.09)	**0.004**
7	47	11	(23.4)	2.35	(0.70–7.72)	2.88	(0.89–9.34)	0.077
8	28	1	(3.6)	0.35	(0.03–3.22)	0.43	(0.48–3.92)	0.460

*PR = Prevalence ratio (of false positive leprosy diagnosis); 95% CI = 95% Confidence intervals

## Discussion

This is one of the first studies from India in recent years, describing the proportion of false positive diagnosis during LCDC campaigns and to assess the effect of appreciative inquiry as an intervention to reduce false positive diagnosis. The study had three important findings. First, in 8 PHCs of 4 districts in Bihar, 303 new leprosy cases were diagnosed during LCDC in September 2016 and a repeat LCDC conducted in February 2018 reduced the number of new cases diagnosed to 118 cases (~62% decline). Second, when a sample of these new cases detected during the two LCDCs was independently validated by a group of experts, the proportion of cases found to be false positive declined from 29.6% in September 2016 LCDC to 23.4% in February 2018 LCDC (6.2% decline). In-between the two rounds of LCDC an appreciative inquiry was conducted by the study investigators involving the district leprosy programme officer and the health care providers of these PHCs. Our inferences based on these study aspects/findings are as follows:

First, there was 62% decline in the total number of cases diagnosed in the 8 PHCs between the two rounds of LCDCs in 2016 and 2018. Though we do not have a control group of PHCs to compare this decline, we had aggregate data on the overall decline in the number of cases detected in the same and neighbouring districts (where AI was not implemented) from the programmatic reports. On an average, the decline in the number of cases was ~42% (range from -92% to +5%). Therefore, we feel that the decline in the number of cases seen in the 8 intervention PHCs is due to the overall decline that can be anticipated between the two rounds of LCDC and is not unique to these 8 PHCs (i.e., it is unrelated to AI).

Second, the adjusted average decline in the proportion of false positive cases between the two rounds of LCDCs was -9% (95% CI: -20% to +1.3%). We feel that this decline is programmatically relevant. However, 95% confidence intervals (CI) are wide and crosses the null value (0%) and therefore we do not have the statistical evidence at the 95% CI level to say that there is conclusive statistical proof about the reduction in the proportion of false positive diagnosis. The wide confidence intervals were due to relatively small sample size (during the February-2018 LCDC) and due to the huge variations in the proportion of false positive diagnosis at the PHC levels. Therefore this should not be termed as “absence of evidence” and result in inaction or rejection of the findings [15]. We therefore estimated the 90% confidence intervals for the adjusted decline and it was -18% to -0.1%. Based on this, we feel that though we do not have statistical evidence for the decline in false positive diagnosis at 95% CI level, we have statistical evidence for this decline at the 90% CI level. We feel that our study provides “proof of concept” that the intervention, has the potential to decrease the false positive cases [16,17].

Third, did AI as an intervention lead to these changes in these 8 PHCs? The ideal study design to provide a confirmatory answer to this question would have been a cluster randomised before and after study. Since we were in a programmatic setup and not a research setup, this ideal study design was operationally not feasible. Even if we were to select a control group of PHCs now, measuring and ensuring that the intervention and control PHCs were almost similar in all aspects except for the intervention in question, is practically impossible. Therefore, we are unable to give a confirmatory answer to this key question. However, our current study design resembles a single arm before and after study design. In 7 out of 8 PHCs the medical officers who had diagnosed the cases in 2016 and 2018 remained the same. They were given an identical refresher training on how to diagnose and treat leprosy before both the LCDC campaigns in 2016 and 2018. However, the only major difference was that, in 2018, they had information on false positive diagnosis. This information was given in a friendly manner using the principles of AI. The health staff who participated in AI meetings quoted that they liked this strategy of change management than the usual hierarchical approach. We therefore believe that AI could have played a role in reducing the false positive diagnosis and the change could have happened through the re-trainings and supportive supervision and monitoring.

Fourth, the most important message for the NLEP from this study is that false positive diagnosis is a major issue during LCDC. This has been highlighted in one of the validation studies done in India during 2004 where 9.4% (95% CI: 7.4%-11.4%) of the cases were found to be wrongly diagnosed as leprosy [18]. And therefore, sufficient measures must be undertaken to address this issue. To our knowledge there are no published studies in the literature since 2004 describing the magnitude of false positive diagnosis during LCDC. Hence, we are unable to compare and contrast our study findings with the false positive diagnosis in other settings or describe the circumstances under which false positive diagnosis is likely to be high or low. Furthermore, our study does not provide information on false negative diagnosis (i.e., the number and proportion of true cases of leprosy missed during the LCDC) which is essential to reduce transmission. These issues have to be explored in future through more operational research studies or validation exercises.

Fifth, the occurrence of false positive diagnosis and false negative diagnosis is due to the “subjectivity” in the diagnosis of leprosy cases due to its dependence on clinical criteria. There are several commentaries/studies on how using clinical criteria can lead to misdiagnosis [19–21]. There are serological tests to assess infection of leprosy that could be used for difficult cases (antibodies against Phenolic glycolipid (PGL-1) Mycobacterium leprae antigen) [22] or use split skin smears [23]. We need to explore this on a programmatic perspective to reduce misdiagnosis. Therefore, in order to reduce the errors in diagnosis, we strongly feel that NLEP must consider making the diagnostic criteria more ‘objective’, introduce more rigorous/comprehensive methods for training of medical officers and/or constitute a committee of two or more trained medical officers at the PHC level to arrive at diagnosis of leprosy. Given the human resource shortages at PHC level in Bihar, we are not sure whether this suggestion is practically feasible or not. Assessing which of these measures will help in reducing misdiagnosis of cases under routine programmatic setting is an area for future research.

Lastly, we strongly believe that the validation exercise conducted by DFIT in the limited number of PHCs helped identify an important operational problem and therefore this needs to be done in all other districts and other states of India. The protocols for validation have been developed by NLEP but the validations are not carried out as envisaged. The NLEP must focus on routine validation exercises in future.

## Conclusion

In conclusion, about one in three cases diagnosed as leprosy during LCDC in 2016 in 8 PHCs of Bihar was found to be false positive. This reduced to one in four cases during the LCDC conducted in February 2018 due to the implementation of AI. Though the decline in proportion of false positive diagnosis is not statistically significant at 95% CI level, we believe the findings are programmatically important.

## Supporting information

S1 STROBE Checklist (DOCX)
